# Supplementary material for: Dissociable cognitive strategies for sensorimotor learning
Source: Nat Commun. 2019 Jan 3;10:40. doi: 10.1038/s41467-018-07941-0 (PMC6318272; doi:10.1038/s41467-018-07941-0)
Supplement: Supplementary file 1 — Supplementary Information [file 41467_2018_7941_MOESM1_ESM.pdf]

Dissociable Cognitive Strategies for Sensorimotor Learning

McDougle & Taylor

Supplementary Figures 1-7

Supplementary References

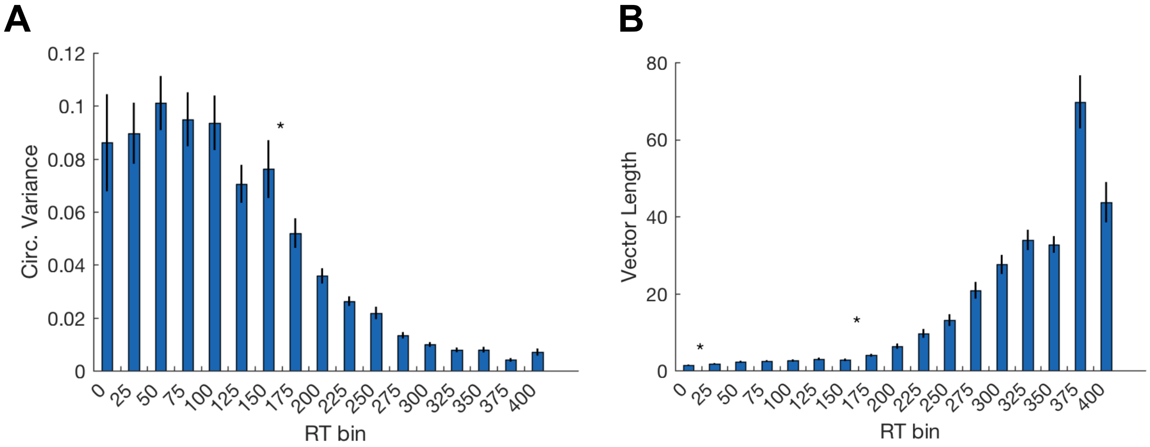

Supplementary Figure 1: *Separating fully random from "random + directed" reaches by RT bin for Experiment 2.* There are several methods for determining when a circular distribution of angles deviates from being a uniform distribution (random) to a non-uniform distribution (having one or more significant clusters of values). Here we show converging results from multiple methods, pointing to the same RT bin (7th) as the critical point where reaches began to be directed rather than random. (**A**) *Circular variance in reach angles*. For each subject, the variance of movement angles at each RT bin was computed and normalized by the number of trials rendered in that bin. Serial *t*-tests were conducted and the first significant test ($\alpha = 0.05$) denoted a "change" in variance. The 7th bin (150-175 ms) was found to be the initial point of significant change ($p = 0.03$). (**B**) *Vector length at each RT bin*. Similar to (A), the vector length at each bin was computed and normalized by number of trials. Serial t-tests revealed the first two significant changes after the 1st bin ($p = 0.03$) and 7th bin ($p = 0.006$), however, due to low numbers of trials in the early bins and known limits of human RT, the 1st bin result was considered spurious. Finally, we also performed a Rayleigh test for uniformity. The Rayleigh test uses vector length to indicate a unimodal deviation from uniformity in a distribution. The first significant deviation was found to be after the 7th bin ($p = 0.02$). Error bars represent 1 s.e.m.

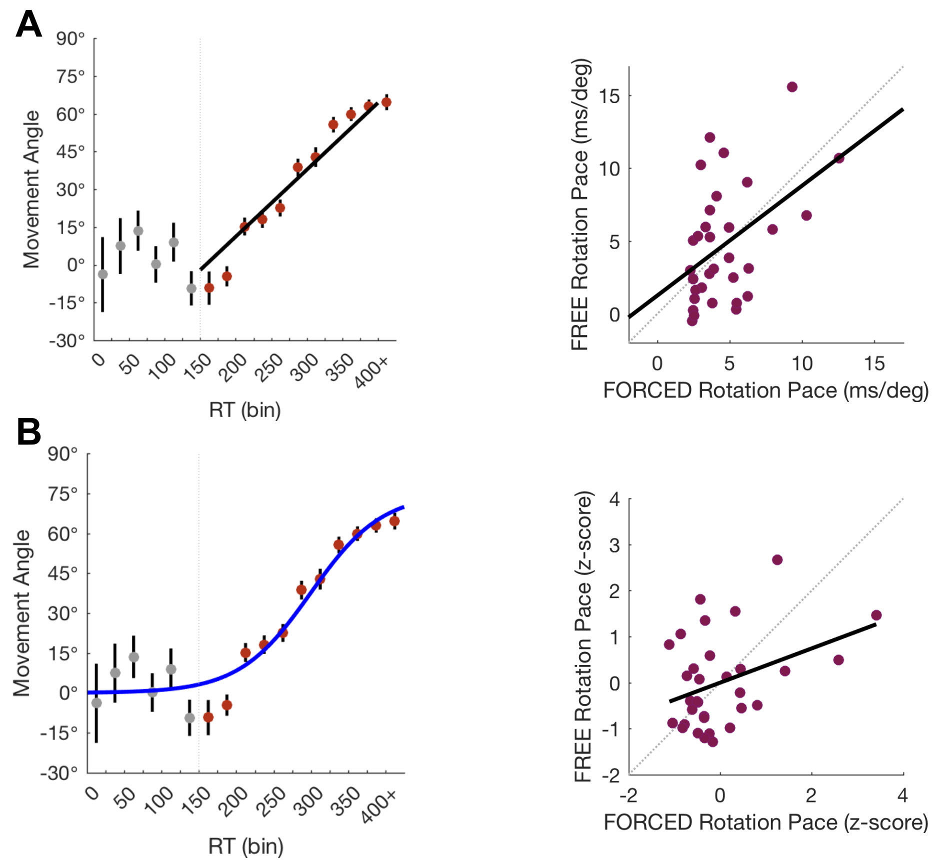

Supplementary Figure 2: *Correlated mental rotation paces between the FREE and FORCED tasks in Experiment 2*. (**A**) Rotation paces for the FREE task were extracted using linear regression (Fig. 4B, main text). For the FORCED task, we used two methods. Here we performed linear regression on a subject's full RT and movement angle data set at all values where RT > 150 ms (left panel). Mental rotation paces were correlated between tasks (right panel; R = 0.46; p = 0.008). (**B**) Given the subtle asymptote in the movement angle data in the FORCED task, we used a second method to derive the mental rotation parameter in the FORCED task. We fit a sigmoid to each subject's full data set (RT > 150 ms; left panel), with free parameters for the offset and rate, fixing the asymptote of the sigmoid function as the subject's mean reach angle in the long RT window trials (1200 ms of target appearance). The mean sigmoid fit (blue) is plotted over the arithmetic mean reach angles. A correlation between z-scored mental rotation paces from the FREE task and FORCED task was weaker though also statistically reliable when using this fitting method (right panel; $R = 0.37$, $p = 0.036$). Error bars represent 1 s.e.m.

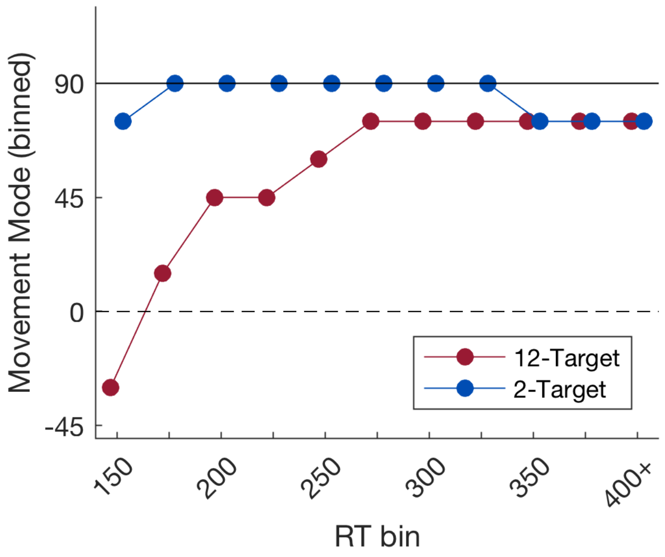

Supplementary Figure 3: *Mode Analysis for Experiments 2 and 3 (FORCED tasks)***.** An alternative explanation for the linear rise in means in the forced task (Fig. 4C, main text) is that it's driven by an averaging artifact. To confirm the presence of "true" non-random intermediate movements, subjects' movements at each RT bin were pooled, binned by 15˚, and a mode was computed. As shown above, the mode of the FORCED task at 12 targets in *Experiment 2* (red dots) matched the trend in their mean data (regression on values above 150 ms bin: p = 0.002). In the 2-target condition, however (*Experiment 3*; blue dots), no significant trend was observed (p = 0.20).

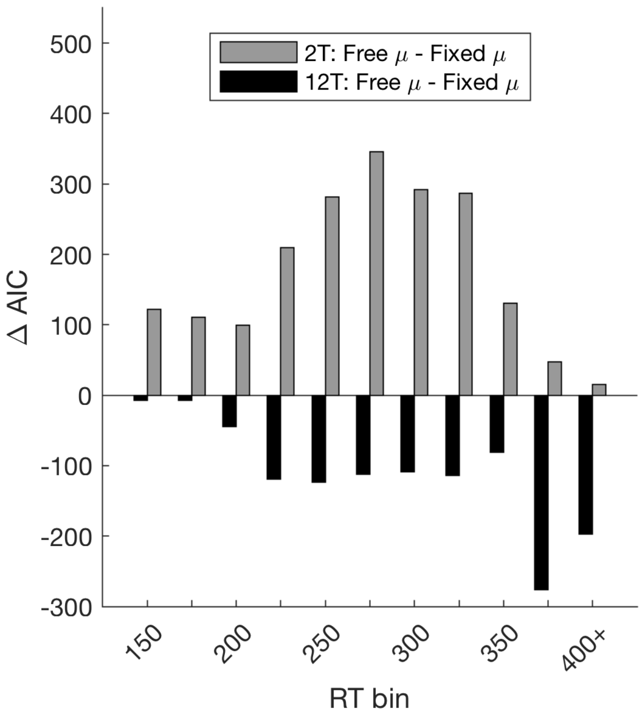

Supplementary Figure 4: *Model comparisons*. ΔAIC values over each RT bin between the Free-μ and Fixed-μ models for the 2-target (grey) and 12-target (black) FORCED-RT experiments. Negative values constitute better fits for the Free-μ model, and positive values constitute better fits for the Fixed-μ model. Recall we found that the 12-target condition (*Experiment 2*) was better fit by the Free-μ model, consistent with using a parametric MR strategy, and the 2-target condition (*Experiment 3*) was better fit by the Fixed-μ model, consistent with a discrete RC strategy. However, for those analyses we summed AIC values across all model fits after and including the critical (7th) RT bin. Here, we compute ΔAIC between models at each RT bin separately. The Free-μ model fit the 12-target group better at every RT bin (ΔAIC range: 6.92 - 276.13). In contrast, the Fixed-μ model fit the 2-target group better at every RT bin (ΔAIC range: 15.69 - 345.46). Thus, consistent with our predictions, subjects in the 12-target condition were likely using a parametric MR strategy whereas subjects in the 2-target condition were likely using a discrete RC strategy.

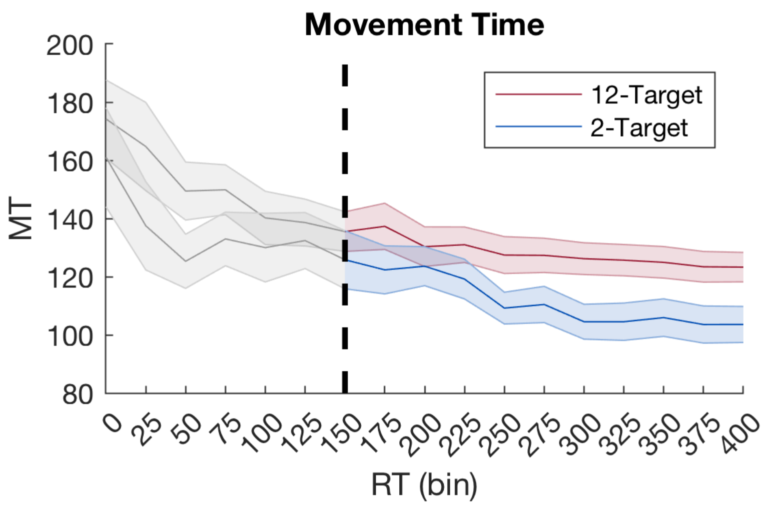

Supplementary Figure 5: *Movement Times (MT) in the two FORCED tasks*. MT in the two conditions across RT bins. MT was numerically lower in the 2-Target condition, though the difference between groups (across all RT bins) was not significant (independent *t*-test, $p = 0.18$).

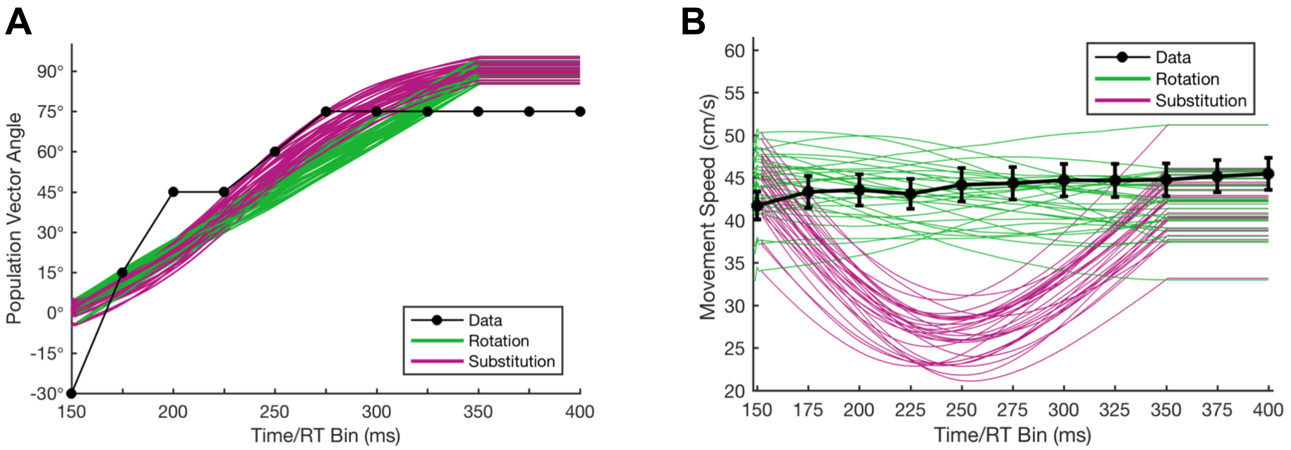

Supplementary Figure 6: *Neural population vector model for movements in Experiment 2 (FORCED Task)*. (**A**) Models of both response substitution and mental rotation predict a shift of the population vector direction from the target (0˚) to the solution (90˚) during the RT. Response substitution predicts a subtle sigmoidal shape relative to the strict linear shape of mental rotation. Neither model appears to capture the logarithmic shape of the data (the mode reach directions). Multiple green and purple lines show the results of 30 different simulations for each model. (**B**) The two models make different predictions concerning movement speed. The mental rotation model, because of its fixed input magnitude, predicts stable movements speeds over the course of mental rotation. However, the response substitution model predicts a clear "sink" in velocity during the substitution of responses, as the vector succumbs to "vote-splitting" between the target and solution (i.e. the population vector's variance increases / its length decreases). Subjects' reach speeds (black dots) were relatively stable across mental rotation RT bins, as predicted by mental rotation. Indeed, mean movement speed continuously rose across RT bins (t-test on regression slopes: $t(31) = 4.28$, $p < 0.001$), arguing against a "ceiling" effect. The response substitution model provides a weaker picture of the movement speed data. Error bars represent s.e.m. Multiple lines show the results of 30 different simulations. See below for modeling details.

We adapted the response substitution model of tuned motor cortical neurons described by Cisek and Scott [1], which they used to argue that response substitution is a more parsimonious interpretation of the neural results of Georgopoulos et al. [2] than mental rotation. We also developed our own mental rotation variant of this model, and compared the behavioral predictions of the two models.

The model of cortical directionally-tuned neurons is formalized as follows [1]:

$$\frac{dx_i}{dt} = \kappa(-x_i + g_i E_i) \qquad (1)$$

$$y_i = [x_i - \Gamma_i] + b_i \qquad (2)$$

where y_i is the simulated activity of M1 neuron i, and x_i reflects the input to that neuron. The parameter k represents a time constant, g_i is a gain parameter, Γ_i is an activity threshold, and b_i is baseline activity (which is set to 0 in this model, see [1] for details). During the simulations, if activity ever goes negative

at any time point, it is reset to a value of 0. The rate of activity growth (Supplementary Equation 1) is driven by the amount of excitatory input, E_i. In the simulations, cosine-tuned cells are given input at the start of the trial, with direction ϕ_j and magnitude a_j, where j indexes the details of the trial (target and goal directions):

$$E_i = \sum_j a_j[\cos(\theta_i - \phi_j) + s_i] \qquad (3)$$

where θ_j is the cell's "preferred" direction and s_i is the offset of the tuning function. Preferred directions of the cells were uniformly distributed from 0 to 360˚. All model parameters (see table below) were set to the values reported in [1]. Moreover, the same number of neurons were simulated (300), though here the simulations were iterated 30 times (due to high noise in the parameter settings) to derive a range of responses.

Finally, rotation trials are simulated for each neuron, and the resulting population vector is computed as follows:

$$\vec{P} = \sum_i \vec{p_i}(y_i - b_i) \qquad (4)$$

where p_i is the unit vector in each cell's preferred direction, which is summed to yield the population vector P. Much research has shown that movement speed is correlated with the motor cortical population vector length [3]; essentially, the more "confident" the population is of the planned movement direction, the faster the resulting movement tends to be. We converted the population vector lengths of both models to movement speeds (in cm/s) by scaling them by an arbitrary scaling parameter with a value of 0.76.

**Parameter Values**	
k	1.3
g	$N(1.2,0.5)$
Γ	$N(0.4,0.2)$
s	$N(0,0.3)$

We modeled neural population activity as a result of either response substitution or mental rotation as follows: For response substitution, two distinct, independent inputs drive neural activity (as in [1]), one reflecting the target direction (e.g. 0˚) and one reflecting the solution direction (e.g. 90˚). For the first 150 ms, input represents solely the target direction, at a maximum magnitude of a = 1, and the magnitude of the solution direction (e.g. 90˚) at the minimum a = 0. During the next 200 ms, these two magnitudes linearly switch values, with the target direction signal decaying to zero and the solution direction signal increasing to 1. The two signals then remain at constant values for the final 150 ms of the simulation.

For mental rotation, only a single input is considered, and its direction is smoothly "rotated" during the RT. Like RS, for the first 150 ms the target direction drives the input at a magnitude of 1 (reflecting the subject processing the target location and anchoring where they need to rotate from). Then, the input angle (ϕ) linearly shifts from 0° to 90° for the following 200 ms, with no change in magnitude. The signal then remains at the solution direction for the remainder of time (150 ms). Finally, for both models, cell recruitment delays were drawn from a uniform distribution from 50 ms to 150 ms.

These two input schedules reflect the logic of response substitution and mental rotation, respectively: In response substitution, a prepotent response to reach toward the target drives activity at first, and is slowly replaced by a new motor plan directed toward the solution. In mental rotation, however, a single plan is directed toward the target and then smoothly rotated toward the solution.

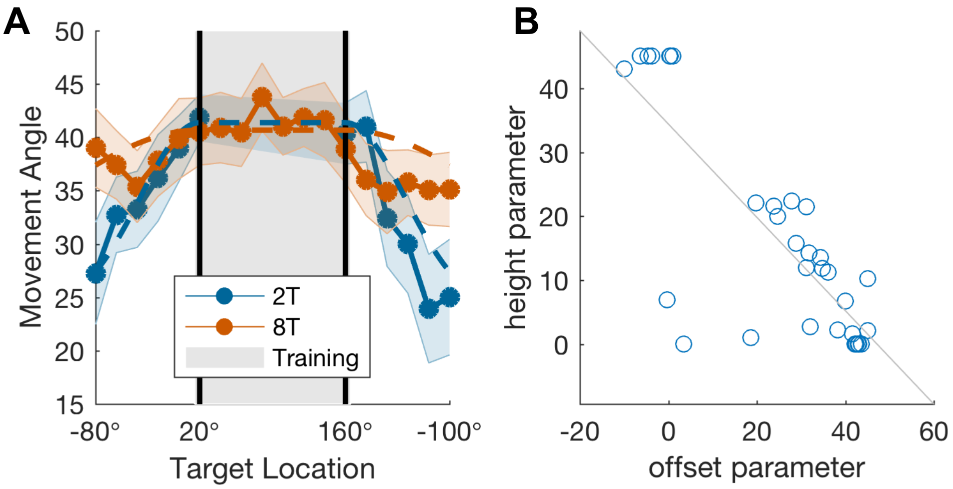

Supplementary Figure 7: *Gaussian fits in Experiment 4*. For *Experiment 4*, we also assessed group differences in generalization by doing a more traditional Gaussian-fitting approach. We fit a composite function that was composed of two half-normals with means at the two respective extreme training targets (rotated in all subjects to 20˚ and 160˚, respectively), with free parameters for the width, height, and offset of the functions (i.e., all three parameters are shared between the two half-normals). Data in the area between the two extreme targets (8T condition) was not modeled. Free parameters were constrained between [0˚ 360˚] for the width, [0˚ 45˚] for the height, and [-5˚ 45˚] for the offset. Fits were performed over 100 iterations per subject with randomized starting parameter values drawn from a uniform distribution on each iteration, minimizing the root-mean-squared error between the modeled function and the reaching data in the generalization block. **A)** Results of the mean fits are displayed as dashed lines. **B)** Critically, because the generalization patterns could be captured by different combinations of the three free parameters, parameter correlations emerged that made interpretation of the results difficult; namely, the offset and height parameters were strongly negatively correlated ($r = -0.80$, $p < 0.0001$). This likely occurred because "flat" generalization patterns can be captured by either a function with a high offset but minimal height and width, or, alternatively, a high width and different combinations of height and offset. We note that while this method did indeed produce the predicted difference in width parameters between the groups, with the 8T group showing a significantly larger width ($t(30) = 2.13$, $p = 0.04$), the fitting procedure was sensitive to different constraints and starting values. Thus, we opted for a more interpretable linear model of the difference in movement angle based on the generalization probe target's distance from the nearest training target (Figure 10C, main text).

Supplementary References

1. Cisek, P. & Scott, S.H. An alternative interpretation of population vector rotation in macaque motor cortex. *Neurosci. Lett.* **272**, 1-4 (1999).

2. Georgopoulos, A.P., Lurito, J.T., Petrides, M., Schwartz, A.B. & Massey, J.T. Mental rotation of the population vector. *Science* **243**, 234-236 (1989).

3. Moran, D.W. & Schwartz, A.B. Motor cortical representation of speed and direction during reaching. *J. Neurophysiol.* **82**, 2676-2692 (1999).
